# The intrinsically disordered distal face of nucleoplasmin recognizes distinct oligomerization states of histones

**DOI:** 10.1093/nar/gkt899

**Published:** 2013-10-08

**Authors:** Isbaal Ramos, Noelia Fernández-Rivero, Rocío Arranz, Kerman Aloria, Ron Finn, Jesús M. Arizmendi, Juan Ausió, José María Valpuesta, Arturo Muga, Adelina Prado

**Affiliations:** ^1^Departamento de Bioquímica y Biología Molecular, Facultad de Ciencia y Tecnología, Universidad del PaísVasco, P. O. Box 644, 48080 Bilbao, Spain, ^2^Unidad de Biofísica (Consejo Superior de Investigaciones Científicas-Universidad del País Vasco/Euskal Herriko Unibertsitatea), Barrio Sarriena s/n, 48080 Leioa Spain, ^3^Centro Nacional de Biotecnología (CNB-CSIC), Darwin 3, Campus de Cantoblanco, 28049 Madrid, Spain and ^4^Department of Biochemistry and Microbiology, University of Victoria, Victoria, British Columbia V8W 3P6, Canada

## Abstract

The role of Nucleoplasmin (NP) as a H2A-H2B histone chaperone has been extensively characterized. To understand its putative interaction with other histone ligands, we have characterized its ability to bind H3-H4 and histone octamers. We find that the chaperone forms distinct complexes with histones, which differ in the number of molecules that build the assembly and in their spatial distribution. When complexed with H3-H4 tetramers or histone octamers, two NP pentamers form an ellipsoidal particle with the histones located at the center of the assembly, in stark contrast with the NP/H2A-H2B complex that contains up to five histone dimers bound to one chaperone pentamer. This particular assembly relies on the ability of H3-H4 to form tetramers either in solution or as part of the octamer, and it is not observed when a variant of H3 (H3C110E), unable to form stable tetramers, is used instead of the wild-type protein. Our data also suggest that the distal face of the chaperone is involved in the interaction with distinct types of histones, as supported by electron microscopy analysis of the different NP/histone complexes. The use of the same structural region to accommodate all type of histones could favor histone exchange and nucleosome dynamics.

## INTRODUCTION

Eukaryotic chromatin is a dynamic protein-DNA complex due to the action of several proteins that modify its structure. Three main factors are involved in this process, namely, (i) chromatin remodelers, which are ATP-dependent protein complexes that promote nucleosome sliding or histone eviction from DNA; (ii) protein complexes that post-transcriptionally modify histones (1); and (iii) histone chaperones, defined as proteins that interact with histones, stimulating their transfer to DNA or to another protein partner without being part of the final complex (2). Histone chaperones are a heterogeneous group of proteins that share the ability to bind histones and are involved in many processes that require chromatin remodeling, such as sperm chromatin decondensation after fertilization (3), nucleosome assembly (4), transcription (5), replication and DNA repair (6). Nucleoplasmin (NPM2 or NP), a member of the nucleophosmin/NP family of histone chaperones, was described in *Xenopus laevis* oocyte nuclei and eggs (7), where it is the most abundant protein (7) and can be found as part of multiprotein complexes (8–10).

The crystallographic structures of the N-terminal core domain (120 amino acids) of NPM2 (11), NPM-like (12) and NPM1 (13) show a high degree of structural homology (14,15). This domain folds into an eight-stranded β-barrel with a jelly roll topology, in contrast to the C-terminal domain (80 residues) that adopts a disordered conformation (16). Based on the X-ray structure of a truncated variant, it has been proposed the existence of three protein surfaces in the homopentamer: the oligomerization surface, the lateral surface that has been proposed as the binding region for histone octamers, and the distal face (11), which contains the acidic tracts A2 and A3 and the nuclear localization signal. Hyperphosphorylation of NP activates its chromatin decondensation activity, most likely through its ability to remove linker histones from DNA (17). Egg NP (eNP) is phosphorylated in at least eight residues located in both protein domains (18). It is worth mentioning that although NP becomes dephosphorylated at the mid blastula transition, the chaperone is present in *Xenopus* cells until early tadpole stages (19,20). The 3D reconstruction of pentameric full-length native eNP and of the eNP/H2A-H2B complex obtained by electron microscopy (EM) suggests that H2A-H2B dimers interact with the C-terminal tails at the highly acidic distal protein face (21). Docking of the available structures into the EM map of this complex and proteolysis data indicates that NP contacts the histone fold and the C-terminal tails of both histones. The NP distal face seems to be a versatile partner, as it also binds linker histones H1 and H5 and linker-related sperm-specific binding proteins (SSBP) (22). Thus, NP as other histone chaperones (5,23–25) can bind several histone ligands, an ability that is probably related to the distinct processes they are involved in. In this context, NP has been proposed to play a role in histone storage in the oocyte preventing their aggregation or inefficient interaction with other partners, decondensation of sperm chromatin after fertilization by removing DNA-bound SSBP, replication licensing and nucleosome assembly in early embryonic cells (7,26). Thus, following the aforementioned argument, NP could interact with different histone partners in these processes.

We characterize in this work the interaction of NP with H3-H4 and histone octamers. We find that both ligands bind to the distal region of the chaperone, as found for H2A-H2B (21). Interestingly, the complexes formed between NP and H3-H4 tetramers or histone octamers are different to that reported previously for the chaperone and H2A-H2B, most likely due to the ability of H3-H4 to form tetramers. Supporting this interpretation is the fact that a H3 variant (H3C110E) that destabilizes H3-H4 tetramers interacts with NP similarly to H2A-H2B (21). The 2D average images of the complexes between NP and H3-H4 tetramers or histone octamers are compatible with two NP pentamers wrapping with their distal faces the histone octamer or H3-H4 tetramer located at the center of the ellipsoidal particle. These data are discussed considering the alternative models proposed for the interaction of NP with histones, and the role that the ability of the chaperone to bind differently H3-H4 tetramers and H2A-H2B dimers might have in nucleosome assembly.

## MATERIALS AND METHODS

### Protein purification

Oocyte NP (oNP) and eNP from *X. laevis* were purified as previously published (16). Recombinant NPs (full-length and two truncated variants that lack the last 50 -ΔC50NP- or 80 -ΔC80NP - residues) were expressed and purified as described (16). Natural source H3-H4 and histone octamers were obtained from chicken erythrocytes chromatin on elution from a hydroxyapatite column (27) and kept in 2 M NaCl until use. Recombinant histones (including mutants H3C110E, H3C110A, H2BT112C, H4T71C) were expressed, purified and reconstituted according to (28). NP concentration was determined by the bicinchoninic acid assay (Sigma) and, unless otherwise stated, is given for its pentameric form. The concentration of natural source histones was determined by absorbance at 230 nm. The extinction coefficient of the H3-H4 dimer in water, ε_230_ = 4.1 cm^2^ mg^−^^1^, was estimated by amino acid analysis using norleucine as standard, and the value used for the histone octamer was ε_230_ = 4.2 cm^2^ mg^−^^1^ (29). Recombinant histones were quantified as described (30), and their concentration given as dimers (H3-H4 and H2A-H2B) or octamer. Molar ratios are expressed as NP pentamer/H3-H4 dimer or histone octamer.

### Antibodies

Purified oNP was injected into rabbits and the resulting polyclonal anti-NP, prepared by Abyntek Biopharma S.L. Bilbao, Spain, was used for immunoprecipitation (IP) and immunoblots. Anti-H3 (ab 1791) and anti-H2B (ab 1790) were from Abcam, Cambridge, England, Rabbit IgG from Santa Cruz Biotechnology, and Pierce Goat anti-Rabbit IgG (H + L) peroxidase conjugated from Thermo Scientific. Dynabeads Protein G from Life Technologies, Oslo, Norway. Anti-NP and IgG were crosslinked to the Dynabeads (0.15 mg/µg of antibody) with BS^3^ (bis[sulfosuccinimidyl]suberate) from Thermo Scientific, Rockford, USA, following the manufacturer protocol.

### Titration of NP with histones—Electrophoresis mobility shift assay

NP (2 µM) was mixed with different histone concentrations and incubated in 25 mM Tris–HCl (pH 7.5), 240 mM NaCl, 2 mM MgCl_2_ (Buffer I) at 25°C for 1 h. Additionally, the same experiments were performed at 150 and 100 mM NaCl, to analyze the ionic strength-dependence of complex formation. As the results were comparable, we use 240 mM NaCl in this study to minimize histone aggregation on dilution of the ligands, stored in 2 M NaCl, in NP-containing buffer. Histone-NP complexes dissociate above 400 mM NaCl (not shown), thus explaining why they similarly associate at 150 and 240 mM NaCl. Native PAGE was carried out in 4–16% PAGE precast Novex Native Bis–Tris gels (Invitrogen), and proteins were stained with Coomassie Brilliant Blue. The stoichiometry of the high molecular mass complex was estimated by 2D electrophoresis and densitometry using known amounts of each protein as standards. Proteins were stained with Zn-Imidazole negative staining after the 4–16% Native-PAGE (31) and with Coomassie Brilliant Blue after the 12.5% SDS–PAGE.

### Sucrose gradients

Gradients (5–20% sucrose) were prepared in buffer I in 12 ml tubes, using the Gradient Master 107 (BIOCOMP, Fredericton, NB, Canada). Increasing concentrations of histones H3-H4 or histone octamers, stabilized in 2 M NaCl, were mixed with NP (0.9 µM) in buffer I. After 1 h at 25°C, the samples were loaded onto the sucrose gradients. Gradients were run on a TST 41.14 swinging bucket rotor (Kontron) at 29 000 rpm for 22 h at 4°C and fractionated after centrifugation. Proteins were precipitated with 20% trichloroacetic acid (TCA) and analyzed by 12.5% SDS–PAGE. Fractions from the gradient peak were also analyzed by 12.5% SDS–PAGE without TCA precipitation, to avoid differences in the acid-induced precipitation of the different proteins that could hamper estimation of their molar ratio in the complexes.

### Analytical ultracentrifugation of NP/histone complexes

Sedimentation velocity runs were performed on a Beckman XL-I analytical ultracentrifuge using an An-55 Al rotor. Samples in buffer I were loaded in double sector cells with aluminum filled Epon centerpieces. Experiments were performed at 40 000 or 44 000 rpm for eNP/histone octamer or eNP/H3-H4 mixtures, during 1.5 h at 7°C. Ultraviolet scans were taken at 230 nm and analyzed by the van Holde and Weischet method (32) using XL-A Ultra Scan II v6.0 software (Borries Demeler, Health Science Center, University of Texas, San Antonio, TX, USA). NP concentration was 0.6 µM. Sedimentation equilibrium experiments were carried out using an An-60 Ti rotor. Samples, containing 0.3 µM NP in buffer I, were loaded on six-hole charcoal-filled Epon 12-mm cells. All runs were carried out at 4°C. Scans were analyzed using XL-A Ultra Scan II v6.0 software (Borries Demeler) using global non-linear least squares curve fitting (33,34). Protein samples were analyzed for equilibrium conditions achieved at different rotor speeds (8000 and 12 000 rpm). The partial specific volumes of the proteins were calculated from their amino acid composition using published values (35).

### Preparation of oocytes and eggs extracts

Hormones injection protocols were as described (36). To prepare oocyte extracts, frogs were anesthetized with MS-222 and sacrificed according to standard and approved protocols [(University of the Basque Country (UPV/EHU), Institutional Animal Care Committee with guidelines from the Spanish Council on Animal Care) (CEBA/51- PO3-01/2010)]. To prepare egg extracts, frogs were handled following approved protocols (CEBA/51- PO3-02/2010). High-speed oocytes (oHSE) and eggs (eHSE) extracts were prepared as described in (37) and (38), respectively, with the following modifications: cytochalasine B was omitted in the buffers, low-speed supernatants were obtained by centrifugation at 8000 rpm (Beckman JS-13.1 rotor, 30 min) and high-speed supernatants by centrifugation at 35 000 rpm (Kontron TST 41.14 rotor, 90 min) at 4°C. The final protein concentration was ∼20 mg/ml.

### Immunoprecipitation

Fifty microliters of oHSE or eHSE were incubated with 5 µg of α-H3 or α-H2B for 2 h at 4°C, and afterwards with 100 µg of polyclonal α-NP or IgG (both crosslinked to Dynabeads Protein G) overnight at 4°C. After magnetic-precipitation, the dynabeads (0.15 mg/µg of antibody) were washed extensively with 20 mM Hepes (pH 7.4), 100 mM NaCl, 5 mM EDTA, 0.05% NP40, boiled with 25 µl of sample buffer and loaded in 18% SDS–PAGE. The gel was run at 120 V at room temperature. Western blots were done as described in (16), and the antibodies were detected with the chemiluminiscence method (Super Signal West Pico Chemiluminescent substrate, Thermo Scientific). The same experiments were repeated with α-NP crosslinked to the Dynabeads but without the initial incubation with anti-histone antibodies. Controls were also prepared by incubating oHSE and eHSE with α-H3 or α-H2B (5 µg/50 µl extracts) and immunoprecipitation (IP) with Dynabeads.

### Limited proteolysis of NP/histone complexes by Trypsin

Partial proteolysis of complexes between eNP and H3-H4 (1/2 molar ratio) or histone octamers (1/0.5 molar ratio), and of the corresponding isolated proteins was carried out in buffer I. eNP or eNP/histone complexes (3.5 µM NP) were incubated at 37°C with trypsin (1/500) (w/w). Aliquots were taken at 30 min, and the reaction was stopped by lowering the pH to 2.5 with acetic acid. GluC (modified Glutamic C-sequencing grade, Princeton Inc.) digestion of the same samples was performed in 240 mM NaCl, 2 mM MgCl_2_, 50 mM Tris–HCl (pH 8.0). Samples were incubated at room temperature with GluC (1/500) (w/w) for 30 and 60 min, the reaction was stopped with SDS–PAGE sample buffer, and the resulting fragments were analyzed by 12.5% Tris-tricine SDS–PAGE.

### Mass spectrometry analysis

Selected tryptic bands were excised manually from the gel and subjected to in-gel tryptic digestion according to (39) with minor modifications (40). LC-MS/MS analysis was done using a Q-TOF Micro mass spectrometer (Waters) interfaced with a CapLC chromatography system (Waters) as described (40). Briefly, digested peptides were loaded onto a Symmetry300 C18 NanoEase Trap (0.18 mm × 23.5 mm, Waters) precolumn connected to an Atlantis dC18 NanoEase (75 µm × 150 mm, Waters) column. Peptides were eluted with a 30 min linear gradient from 10 to 60% acetonitrile. Data-dependent MS/MS acquisitions were performed on precursors with charge states of 2, 3 or 4 over a survey m/z range of 400–1500. Collision energies were varied as a function of the m/z and charge state of each peptide. Spectra were processed and searched against SwissProt database using ProteinLynx Global Server 2.1 (Waters) using standard searching parameters.

### EM methods

#### Gradient fixation method

eNP/histone complexes were prepared for EM by the gradient fixation (GraFix) technique as described in (41) with minor changes. Gradients (10–30% glycerol) were prepared in 25 mM Hepes, 240 mM NaCl, 2 mM MgCl_2_ (pH 7.5), using the Gradient Master 107 (BIOCOMP, Fredericton, NB, Canada), the 30% glycerol solution containing 0.15% glutaraldehyde. eNP was diluted to 2.4 µM in the aforementioned buffer and mixed with histones to obtain the eNP/H3-H4 (1/2) and eNP/octamer (1/0.5) mixtures, which were incubated 1 h at room temperature. Histones were diluted from stock solutions containing 2 M NaCl in the presence of eNP so that the final NaCl concentration was 0.24 M. Samples loaded in the glycerol fixation gradients were run on a SW60TI swinging bucket rotor (Beckman) at 35 000 rpm for 16 h at 4°C and fractionated after centrifugation. Complex migration peaks were located by 12.5% SDS–PAGE analysis of the fractions.

#### EM and image processing

Samples (either NP/H3-H4 or NP/octamer complexes) were applied onto carbon-coated copper grids previously glow-discharged and stained with 2% uranyl acetate. Images were taken in a FEI Tecnai G^2^ FEG200 electron microscope operated at 200 kV and using a Gatan side-entry cryo-holder and recorded in a CCD-camera at a x67000 nominal magnification with sampling window corresponding to 4.42 Å/pixel for all the specimens. Individual particles were manually selected using XMIPP software package (42). Image classification was performed using a free-pattern maximum-likelihood multi-reference refinement (43). Homogeneous populations were obtained (493 and 412 particles for the eNP/octamer and eNP/H3-H4 complexes, respectively), and averaged for a final 2D characterization.

#### Fluorescence resonance energy transfer experiments

H2BT112C and H4T71C were labeled with Alexa 488 and Alexa 350 (Invitrogen) respectively, at a protein/fluorescent probe molar ratio of 1/5, in 20 mM Tris–HCl (pH 7.4), 1 mM TCEP (Tris(2-carboxyethyl) phosphine hydrochloride), 6 M guanidine hydrochloride and incubated overnight at 4°C as indicated (44). Labeled histones were mixed with H2A and/or H3C110A to reconstitute the corresponding complexes (28). Reconstituted dimers, tetramers and octamers were purified by size exclusion chromatography. The labeling efficiency for H2BT112C was 75%, and for H4T71C, it was 35–40%. Fluorescence resonance energy transfer (FRET) measurements were recorded in a Fluorolog-3 spectrofluorometer (Horiba Jobin Yvon) with 2/2 nm slit widths. eNP/histone complexes and corresponding controls were incubated for 1 h at 25°C in 25 mM Tris–HCl (pH 7.5), 240 mM NaCl, 2 mM MgCl_2_. In all cases, NP concentration was 2 µM.

## RESULTS

### eNP/H3-H4 or histone octamers complexes are different from those made by eNP and H2A-H2B

**Figure 1.**
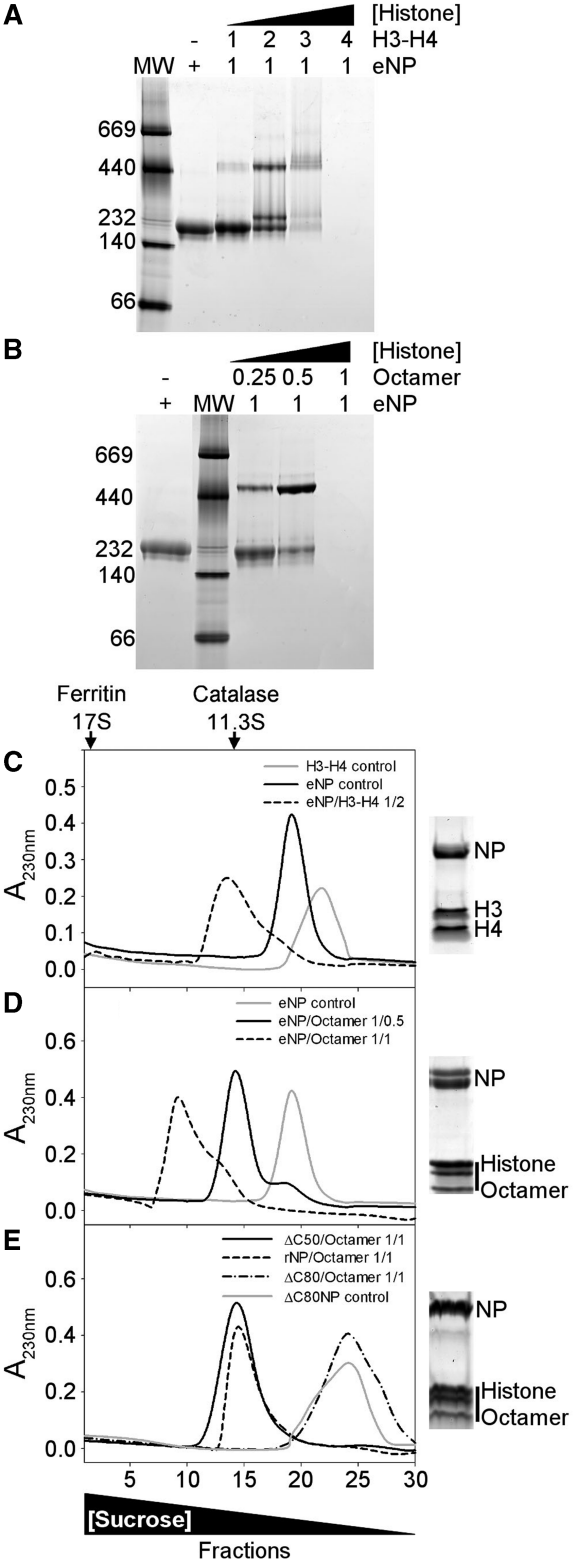
Analysis of NP/H3-H4 and NP/histone octamer complexes. (**A**) eNP was titrated with H3-H4 and complex formation, at the indicated eNP/H3-H4 molar ratios, followed by 4–16% Native-PAGE. The MW markers position (left lane) and a triangle showing the increase in H3-H4 concentration are also shown. (**B**) eNP was mixed with increasing amounts of histone octamer, and the samples were analyzed by Native–PAGE. Other details as in (A). (**C**) Sucrose gradient fractionation of the eNP/H3-H4 1/2 complex. Absorbance at 230 nm of the gradient fractions of H3-H4 control (gray solid line), eNP control (black solid line), and eNP/H3-H4 1/2 complex (dashed line). SDS–PAGE of fraction 13 of the eNP/H3-H4 complex. (**D**) Sucrose gradient fractionation of NP/octamer complexes. A_230_ of the gradient fractions corresponding to eNP control (gray solid line), eNP/octamer 1/0.5 (black solid line), and eNP/octamer 1/1 complex (dashed line). SDS–PAGE of fraction 14 of the eNP/octamer (1/0.5) complex. (**E**) Sucrose gradient fractionation of rNP mutants/octamer complexes. A_230_ of the fractions corresponding to ΔC50NP/octamer 1/1 (black solid line), rNP/octamer 1/1 (dashed line), ΔC80NP/octamer 1/1 (dashed-dotted line) complexes, and to ΔC80NP alone (gray solid line). SDS–PAGE of fraction 14 of the rNP/octamer (1/0.5) complex. The triangle in the lower part of the figure shows changes in sucrose concentration. The migration of two control proteins, catalase (232 kDa, 11.3 S) and ferritin (440 kDa, 17 S), is marked in the upper part of panel (C).

The interaction between eNP and histones was first tested by native PAGE (Figure 1A and B). The electrophoretic mobility of eNP and eNP/H3-H4 complexes obtained at different molar ratios indicates that complex formation gives rise to low and high molecular weight bands, showing the existence of at least two major distinct assemblies (Figure 1A, lanes 2–5). As migration in native–PAGE also depends on the shape and charge of the complex, this experimental method cannot be used to accurately determine its molecular mass. Although an estimation of the eNP/histone stoichiometry of the different complexes by this method is not straightforward, these data indicate that the interaction of NP with H3-H4 at low molar ratios (i.e. less than 1/4) results in a high molecular weight complex (apparent MW ≈ 500 kDa), similar to that described previously (45), which is not seen with H2A-H2B (21). At eNP/H3-H4, molar ratios higher than 1/3 the complex does not enter into the gel, most likely due to a charge neutralization effect (Figure 1A). The stoichiometry of the high molecular weight band was estimated to be 1 NP pentamer/2.1 ± 0.1 H3-H4 dimers by 2D electrophoresis and densitometry of the corresponding bands using known amounts of the same proteins as standards (Supplementary Figure S1). When eNP is titrated with histone octamers, the intensity of the band corresponding to free NP decreases with increasing histone concentration, and a high molecular mass band (apparent MW ≈ 500 kDa) appears at eNP/octamer molar ratios lower than 1/1 (Figure 1B). As previously mentioned for H3-H4, complexes containing higher amounts of octamers do not enter into the gel (Figure 1B), most likely due to charge neutralization (46). This interpretation is supported by the shift of the pI values of the complexes to neutral pH, as compared with those of eNP (Supplementary Figure S2).

The same samples were analyzed by sucrose (5–20%) gradient ultracentrifugation (Figure 1C and D). Samples containing NP/H3-H4 at a 1/2 molar ratio showed one broad main peak that sediments faster than free eNP or H3-H4 and contains both eNP and H3-H4 (Figure 1C). The broad range of differently phosphorylated species of eNP can be resolved as two discrete bands in SDS–PAGE (Figure 1C), as previously reported (47). Interestingly, this complex sedimented faster than those obtained with the same relative amount of H2A-H2B (21), suggesting that they are different. A similar behavior is observed for samples containing eNP and histone octamers at 1/0.5 and 1/1 molar ratios (Figure 1D). The asymmetry of the 1/1 complex profile, in contrast to that of the 1/0.5 sample, suggests that it can associate to form larger and heterogeneous assemblies. The stoichiometry estimated for the NP/octamer (1/0.5) complex from the gradient peak fractions or by 2D electrophoresis (Supplementary Figure S1) was similar (NP pentamer/octamer 1/0.50 and 1/0.54 ± 0.1, respectively), suggesting that most of the chaperone and histones are engaged in complex formation. It is worth mentioning that under these experimental conditions, i.e. 0.24 M NaCl, the free octamer dissociates.

The relevance of the phosphorylation state of NP and the role of the C-terminal domain in eNP/octamer complex formation has also been investigated (Figure 1E). For this purpose, three different variants of recombinant, non-phosphorylated NP were used instead of hyperphosphorylated eNP: full-length NP (rNP) and two truncated mutants in which the C-terminal domain was partially (ΔC50NP) or completely (ΔC80NP) deleted (16). From the sedimentation profiles of the complexes, it can be concluded that the phosphorylation state of NP is not essential for NP/octamer complex formation *in vitro*, as rNP and ΔC50NP also bind the octamer (Figure 1E). In contrast, the sedimentation profile of the ΔC80NP/octamer sample overlaps with that of free ΔC80NP, pointing to the ‘tail’ domain as an essential factor to stabilize the complex (Figure 1E). In agreement with these results, native–PAGE shows that the interaction of rNP and ΔC50NP, but not ΔC80NP, with H3-H4 or histone octamers gives rise to the high molecular weight complex (Supplementary Figure S3). When the gel shift experiments were repeated at lower salt concentration, i.e. 100 or 150 mM NaCl, the ΔC80NP variant also forms high molecular weight complexes with H3-H4 and histone octamers (Supplementary Figure S4), in agreement with previously published data (11,12). However, these complexes might be different from those observed with rNP and ΔC50NP, as they do not show the expected decrease in molecular mass associated with the deletion. This difference is clearly observed for the complexes of rNP and ΔC50NP with either H3-H4 or histone octamers (Supplementary Figure S4, panels C and D). However, we cannot rule out that the sensitivity of native electrophoresis to shape and charge could also contribute to the observed differences in molecular mass.

**Figure 2.**
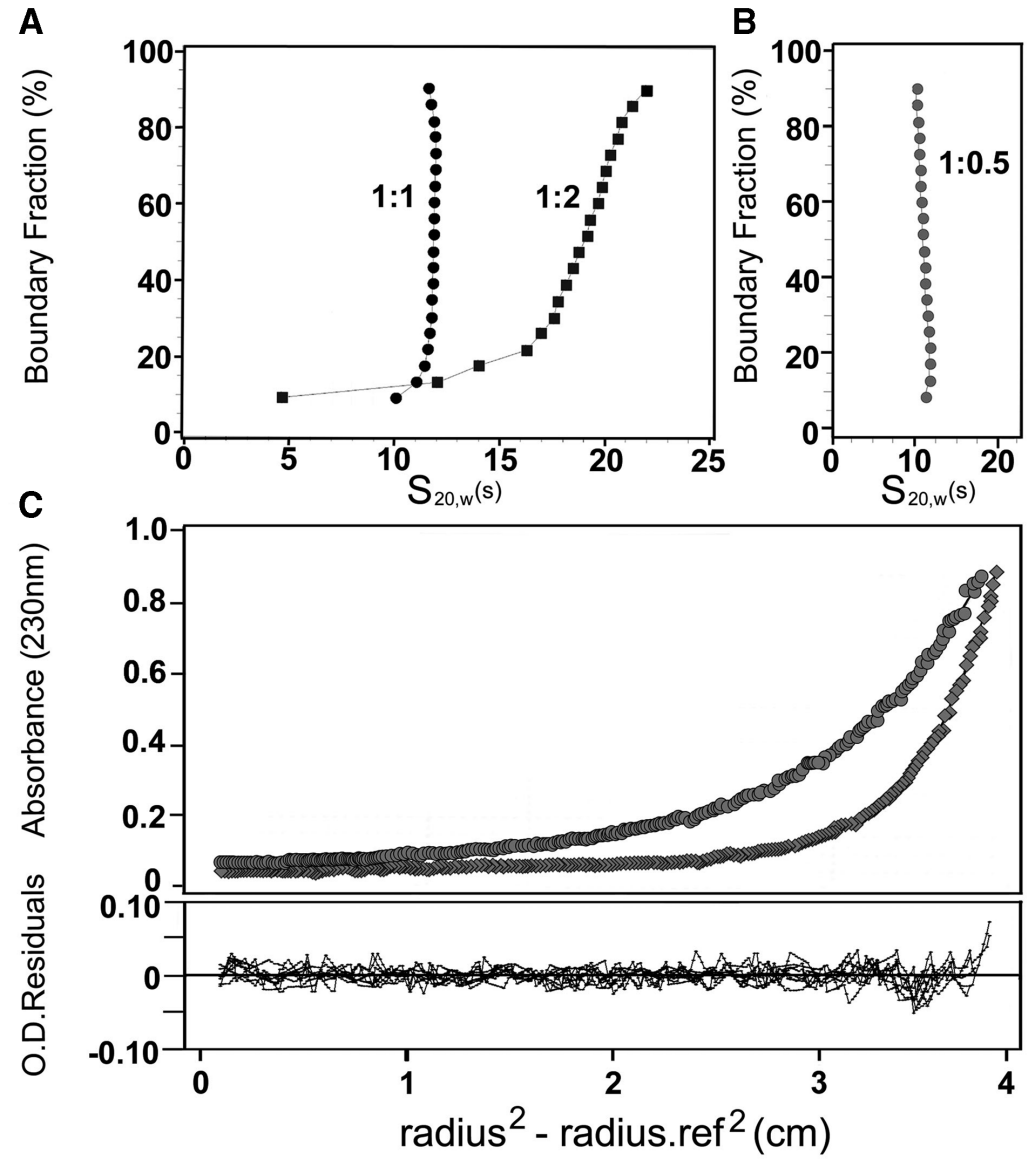
Analytical ultracentrifugation analysis of NP/histone complexes. (**A**) Sedimentation velocity analysis of eNP/H3-H4 complexes at 1/1 and 1/2 molar ratios, and (**B**) of the eNP/octamer (1/0.5 molar ratio) complex. (**C**) Sedimentation equilibrium analysis of the eNP/octamer (1/0.5 molar ratio) complex. The top panel shows the absorbance at 230 nm as a function of the square of the radial distance of the sample at any position within the cell (r) minus the square of the radial position at a reference point (r_0_) (r^2^-r_0_^2^). The continuous lines in these plots were obtained by fitting the experimental data to a single ideal species with Mr of ∼307 kDa. The bottom plot shows X^2^ residuals as a function of r-r_0_^2^ for the best fit (solid lines). The run was performed at 4°C and 8000 (circles) and 12 000 (diamonds) rpm.

To further characterize these complexes, they were analyzed by analytical ultracentrifugation (Figure 2). Samples were prepared as described earlier in the text, by diluting stock histone solutions in 2 M NaCl in the presence of eNP to achieve a final chaperone and NaCl concentrations of 0.6 µM and 0.24 M, respectively. Under these experimental conditions, native H3-H4 and H2A-H2B display sedimentation coefficients (s_20,w_) in the 1.6–2.0 S range, whereas eNP forms a stable pentamer with a sedimentation coefficient of 6.04 S (21). The sedimentation velocity analysis of NP/H3-H4 mixtures at a 1/1 molar ratio shows a narrow distribution of s_20,w_, with a midpoint value of 11 S (Figure 2A), indicating that the complex is homogeneous. At a NP/H3-H4 molar ratio of ½, the distribution is broader, with 65–70% (after correction for the different extinction coefficients of NP and histone dimers) of the sample sedimenting with a s_20,w_ centered ∼19 S, and the rest sedimenting as free eNP and eNP/H3-H4 complexes of very high s_20,w_. The presence of these aggregates, which are not detected in sucrose gradients, could reflect that the complexes are not stable, their components being able to dissociate and associate into larger assemblies. Despite the heterogeneity, the s_20,w_ values are significantly larger than those obtained for eNP/H2A-H2B complexes formed under similar experimental conditions (21), revealing differences between them. At eNP/H3-H4, molar ratios higher than 1/2 the sample aggregates precluding its analysis, in contrast to what was observed for eNP/H2A-H2B mixtures (21). Sedimentation velocity analysis of the eNP/octamer (1/0.5) mixture reveals a homogeneous complex with a s_20,w_ of 12.1 S (Figure 2B). The molecular mass of this complex, estimated by equilibrium sedimentation, is 307 kDa (Figure 2C), suggesting that two eNP pentamers could associate with one histone octamer.

### Formation of the high molecular weight complex depends on H3-H4 tetramerization

**Figure 3.**
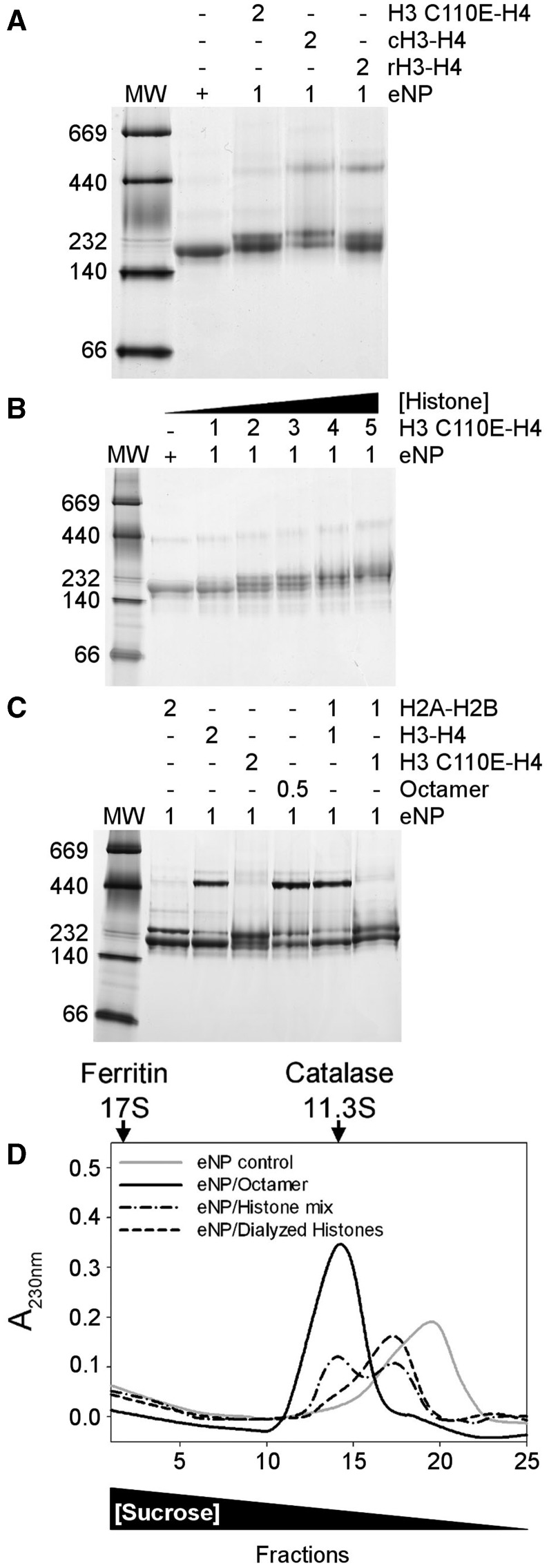
NP binds differently H3-H4 dimers and tetramers. (**A**) Comparison of the complexes formed by eNP and recombinant H3C110E-H4, chicken H3-H4 (cH3-H4), or recombinant H3C110A-H4 (rH3-H4) at a 1/2 molar ratio. (**B**). Titration of eNP with recombinant H3C110E-H4, as seen by Native–PAGE. eNP control and eNP/H3C110E-H4 complexes at increasing histone concentration. (**C**) Comparison of the following eNP/histone complexes: eNP/H2A-H2B 1/2; eNP/H3-H4 1/2; eNP/H3C110E-H4 1/2; eNP/octamer 1/0.5; eNP/H2A-H2B/H3-H4 1/1/1; and eNP/H2A-H2B/H3C110E-H4 1/1/1. In the last two samples, the histone components were added simultaneously from 2 M NaCl stock solutions to NP containing samples. In all panels, the molar ratios of the different eNP/histone complexes are indicated in the upper part of the gels. (**D**) Sucrose gradient fractionation of eNP control (gray solid line), eNP/octamer 1/0.5 complex (black solid line), eNP/H2A-H2B/H3-H4 1/0.5/0.5 complex (black dashed-dotted line), and eNP/H2A-H2B/H3-H4 1/0.5/0.5 complex prepared using histones extensively dialyzed against buffer containing 0.24 M NaCl (black dashed line). The triangle in the lower part of the figure shows changes in sucrose concentration. The migration of the control proteins catalase (232 kDa, 11.3S) and ferritin (440 kDa, 17S) is marked with arrows in the upper part of panel D.

The difference between the apparent molecular masses of eNP/H3-H4 and eNP/H2A-H2B complexes might be due to the interaction of the chaperone with H3-H4 tetramers, an oligomerization state that histones H2A-H2B do not sample. To validate this hypothesis, the interaction between eNP and the H3C110E-H4 variant, which shows an oligomerization equilibrium shifted towards the dimer due to the inclusion of a charged residue at the H3-H3 tetramerization interface (48), was characterized. First, we proved that complex formation between eNP and chicken or recombinant H3-H4 gives rise to the same band pattern (Figure 3A). Second, we compared these complexes with those formed by eNP and H3C110E-H4, a histone variant that is mainly dimer in solution (Figure 3A). When the dimeric variant is used, the high molecular mass complex is hardly detected even at higher histone concentrations (Figure 3B), and instead bands that resemble the complexes described for eNP and H2A-H2B (21) are observed (Figure 3C). These data suggest that the high MW complex comes from the interaction between eNP and H3-H4 tetramers either free in solution or forming part of the octamer (Figure 3C). When the isolated components of the histone octamer are diluted from 2 M NaCl in the presence of eNP, the chaperone binds them forming the high molecular weight complex (Figure 3C). This is not the case when the same mixture is performed with H3C110E that impairs H3-H4 tetramer formation (Figure 3C). The same behavior is observed when complexes are prepared in 150 mM NaCl (Supplementary Figure S5). Moreover, when the H2A-H2B/H3-H4 mixture is extensively dialyzed against 0.24 M NaCl buffer, conditions that favor H3-H4 dimerization, the amount of the high MW complex is negligible (Figure 3D). The sedimentation profile of this sample resembles that of eNP/H2A-H2B complexes (21), suggesting that the chaperone interacts mainly with histone dimers generated on octamer/H3-H4 tetramer dissociation.

### Identification of histones that co-immunoprecipitate with NP

**Figure 4.**
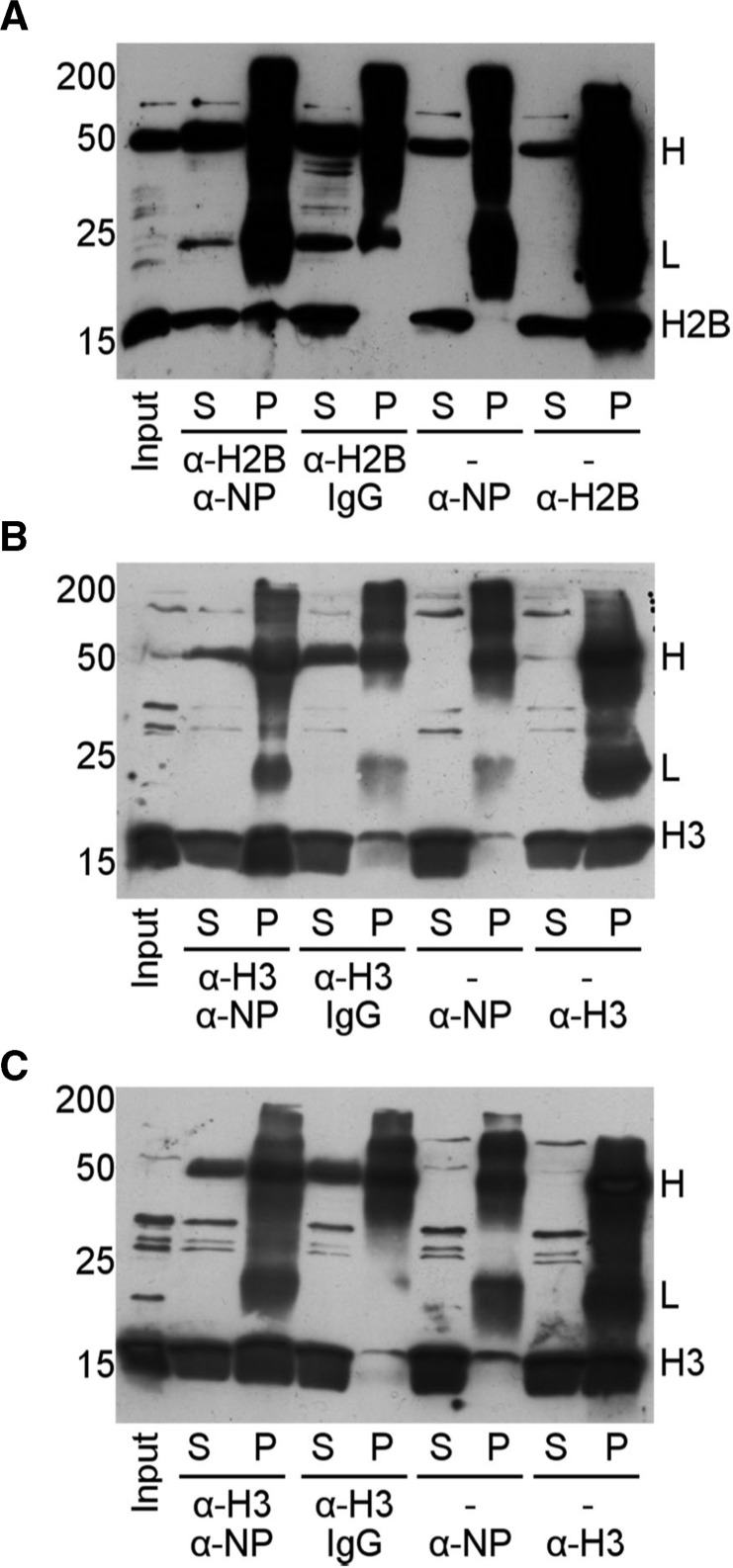
NP/core histone complexes are present in *Xenopus* oocyte and egg extracts. IP of core histones with α-NP. *Xenopus* oocytes (**A** and **B**) and eggs (**C**) extracts were incubated with α-H2B or α-H3 before immunoprecipitating the complexes with α-NP or IgG crosslinked to the Dynabeads. The supernatants (S) and pellets (P) of the IPs were immunoblotted for α-H2B (A) or α-H3 (B and C). Additionally, IP of H2B (A) or H3 (B and C) with α-NP alone was also analyzed without the initial incubation with anti-histone antibodies. The low efficiency under these experimental conditions suggests that the complexes are unstable. Finally, IP of H2B or H3 with their respective antibodies is shown in the right lanes of the panels. The positions of the MW markers (18% SDS–PAGE), of the antibody H and L chains, and of H2B and H3 are indicated.

The interaction of NP with H3-H4 *in vivo* remains yet controversial (9,49,50). Therefore, our next goal was to isolate these complexes by IP and identify their components using *Xenopus* oocytes and eggs extracts. Both systems have been extensively used to reconstitute chromatin remodeling events (36). In agreement with previous studies (36,49), the amount of core histones (H2A-H2B and H3-H4) immunoprecipitated by our polyclonal NP antibody was low (Figure 4), most likely due to the unstability of the complexes that do not withstand the IP procedure (49). Therefore, following published protocols (49), the same IP experiments were carried out incubating first the extracts with α-H3 or α-H2B, to stabilize the complexes, and then immunoprecipitating them with α-NP crosslinked to the Dynabeads (Figure 4). The IP efficiency increased significantly, and both types of core histones, H2B (Figure 4A) and H3 (Figure 4B and C), were found in the complexes obtained from oocytes (Figure 4A and B) or eggs (Figure 4C) extracts. The control of non-specific binding using IgG crosslinked to the Dynabeads showed that the amount of immunoprecipitated histone was negligible (Figure 4). These data demonstrate the existence of complexes containing NP and both types of core histones, H2A-H2B and H3-H4, but however they do not allow to determine whether NP/octamer complexes are present in these extracts.

### Limited proteolysis of the eNP/H3-H4 and eNP/octamer complexes

**Figure 5.**
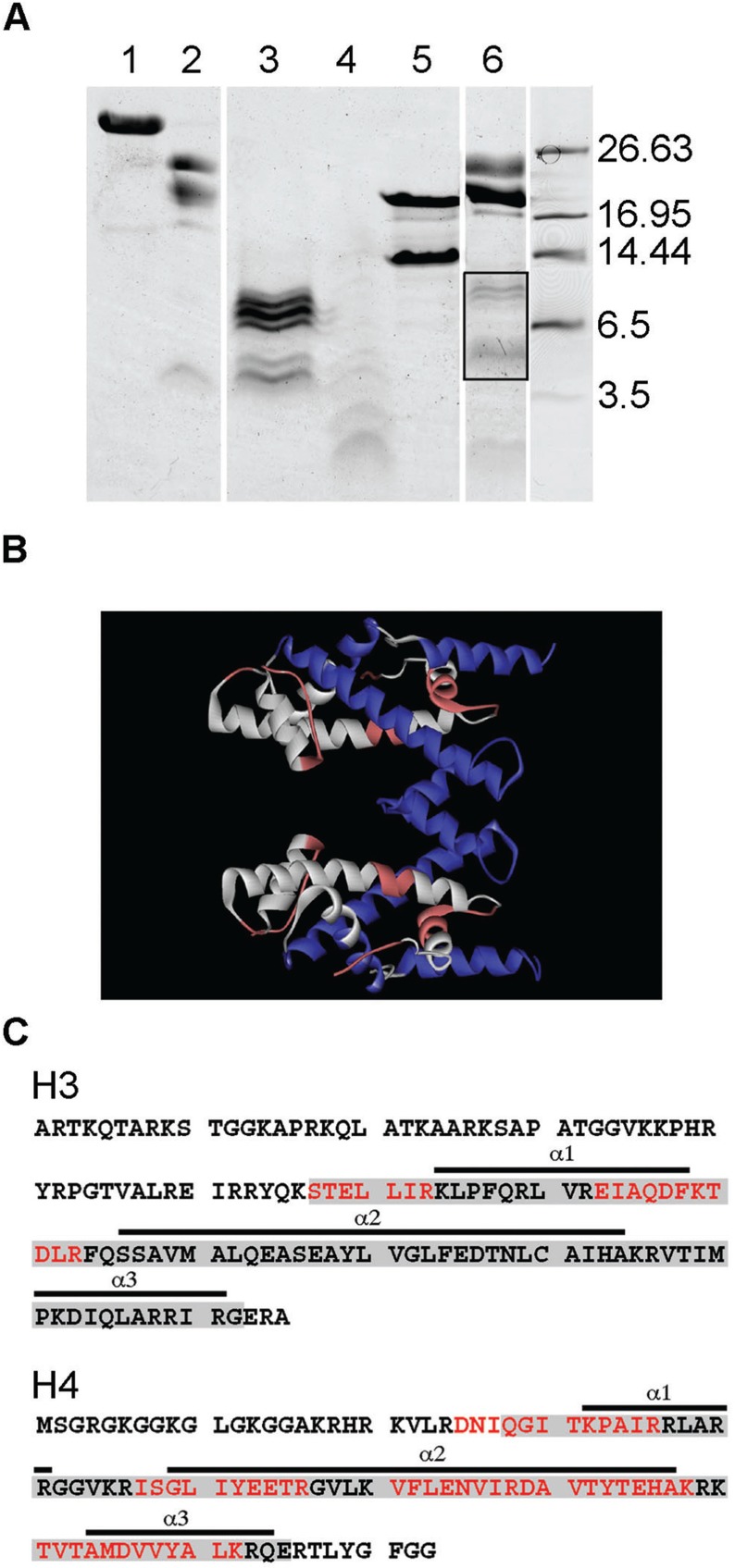
Mass spectrometry analysis of the tryptic peptides obtained from eNP/H3-H4 complexes. (**A**) 12.5% Tris-Tricine gel electrophoresis of the tryptic peptides obtained from eNP/H3-H4 complexes (1/2 molar ratio). The peptides from the bands highlighted within the box were analyzed by MS/MS. 1, eNP control; 2, eNP digested in 0.24 M NaCl; 3, H3-H4 digested in 2 M NaCl; 4, H3-H4 trypsinized in 0.24 M NaCl; 5, H3-H4 control; 6, eNP/H3-H4 1/2 complex digested in 0.24 M NaCl. All samples were incubated with trypsin during 30 min. (**B**) Structure of the H3-H4 tetramer as found in the octamer (entry 1TZY in PDB). Shown in white are the tryptic peptides identified by mass spectrometry from H3 (blue) and H4 (red). (**C**) Amino acid sequence of H3 (accession number 0806228A) and H4 (accession number U37576.1) showing the HFD (gray boxes) and its three helixes (underlined). The peptides protected in the complex (white in panel B) are highlighted in red.

To gain further insights into the regions of these basic ligands that could interact with NP, complexes obtained under conditions that give rise to the high MW assemblies, i.e. diluting the histones from 2 M NaCl solutions, were subjected to trypsin treatment, and the resulting fragments were analyzed by MS. As controls, isolated eNP (Figure 5A; lanes 1 and 2) and H3-H4 (Figure 5A; lanes 3–5) were also digested. Data show, as previously found for H2A-H2B (21), that histones were extensively digested at low ionic strength, i.e. 0.24 M NaCl (Figure 5A, lane 4), whereas at 2 M NaCl, stable fragments were observed (Figure 5A, lane 3). The salt-induced protection against protease cleavage is most likely due to the stabilization of the histone fold that reduces the accessibility of potential protease cleavage sites (51,52). eNP/H3-H4 complex formation protects histone fragments from proteolysis (Figure 5A; lane 6). The molecular mass (Figure 5A) and MS/MS analysis (Table 1) of the tryptic fragments between 4 and 11 kDa indicate that they are similar to those seen after protease treatment of the isolated histones in 2 M salt (Figure 5A, lanes 3 and 6). The sequence of these fragments, all of them coming from H3-H4, indicates that H3L1, most of H4L2 and H4 α1, α2, α3 are protected against protease attack when complexed with eNP (Figure 5B and C). The C and N-terminal histone tails, rich in Lys and Arg residues, are difficult to detect by mass spectrometry after trypsinolysis. These data also suggest that the H3-H3 four helix bundle involved in H3-H4 tetramerization (H3L2, H3 α2, α3) and H4L1 are not protected in the complex, and that the eNP/H3-H4 interacting surface is formed mainly by the H4 histone fold, as it has been described for other H3-H4 histone chaperones (24,53).

**Table 1.** Peptides protected from trypsin digestion in the complexes, identified by MS

Histone	NP/octamer 1:0.5	NP/H3-H4 1:2
H2A	22–33; 37–56; 83–120	
H2B	34–43; 47–72; 80–86; 93–125	
H3	41–49; 57–83	57–63; 73–83
H4	25–36; 47–78; 80–93	25–36; 47–56; 61–78; 81–92

**Figure 6.**
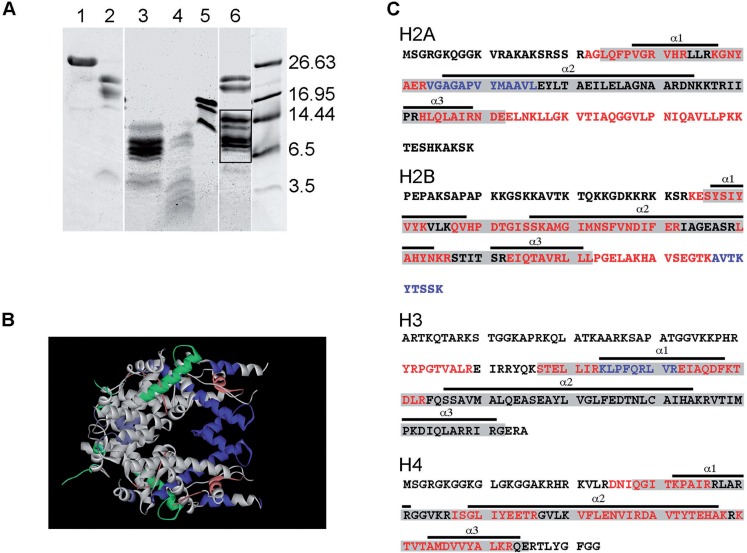
Mass spectrometry analysis of the tryptic peptides obtained from eNP/octamer complexes. (**A**) 12.5% Tris–Tricine gel electrophoresis of the tryptic peptides obtained from eNP/octamer (1/1 molar ratio) complexes. The peptides from the bands highlighted within the box were analyzed by MS/MS. 1, eNP control; 2, eNP digested in 0.24 M NaCl; 3, octamer trypsinized in 2 M NaCl; 4, octamer digested in 0.24 M NaCl; 5, octamer control; 6, eNP/octamer 1/0.5 complex digested in 0.24 M NaCl. All samples were incubated with trypsin for 30 min. (**B**) Structure of the histone octamer (entry 1TZY in PDB). Shown in white are the tryptic peptides identified by mass spectrometry for H2A (green), H2B (turquoise), H3 (blue) and H4 (red). (**C**) Amino acid sequence of H2A (accession number P70082), H2B (accession number P02279), H3 (accession number 0806228A) and H4 (accession number U37576.1) showing the HFD (gray boxes) and its three helixes (underlined). The peptides protected in the complex (white in panel B) are highlighted in red. Additional information obtained using GluC for sample digestion is highlighted in blue.

The tryptic fragments generated on proteolysis of the eNP/octamer complex and the corresponding controls are shown in Figure 6A. As described above for H3-H4, high salt concentration protects the histone fold of the octamer components (Figure 6A, lane 3) (51,52), as it does eNP/octamer complex formation (Figure 6A, lane 6). MS/MS analysis of the protected fragments reveals, with a different sequence coverage for each histone type (44% -H2A-, 54% -H2B-, 20% -H3- and 56% -H4), that the interaction with eNP protects the histone fold of H2A, H2B and H4, and the C-terminal tails of H2A and H2B (Table 1; Figure 6B and C). Therefore, it could be that in the eNP/octamer complex, the H4-H2B four-helix bundle that forms the dimer-dimer interacting region is protected, in contrast to the H4-H2A docking patch and the H3-H3 four helix bundle that are also needed to stabilize the octamer. It should be kept in mind that protection of NP-bound histones against proteolysis may be due to a direct interaction of these ligands with the chaperone that could hinder cleavage sites, and/or to complementary ionic interactions in the complex that would compact histone structure, as found in 2 M NaCl.

### The eNP/octamer and eNP/H3-H4 complexes are formed by two opposed NP pentamers

**Figure 7.**
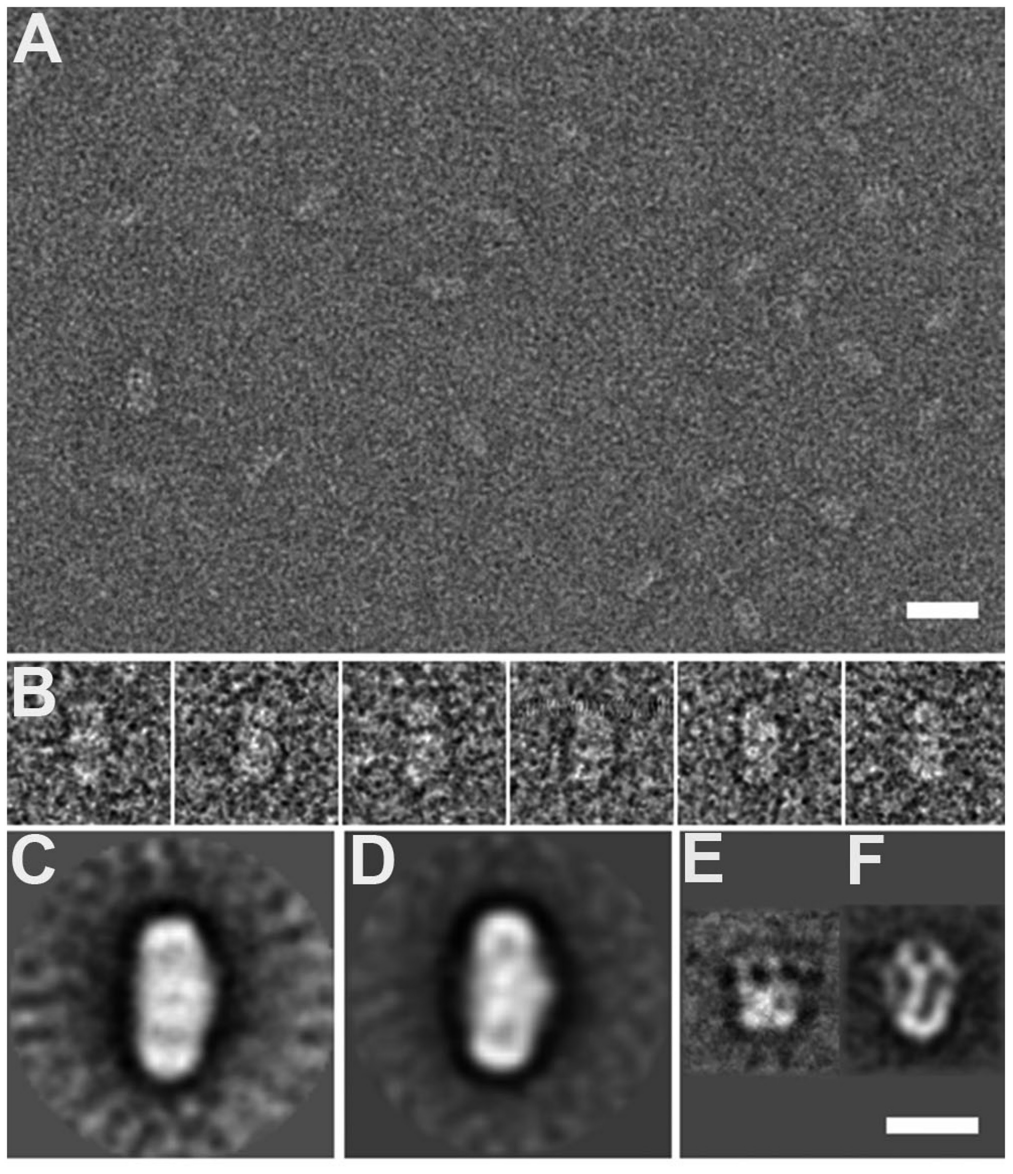
EM of the eNP/octamer and eNP/H3-H4 complexes. (**A**) An EM field of eNP/octamer complexes revealing their ellipsoidal shape. (**B**) A gallery of particles of the eNP/H3-H4 1/2 complex showing its symmetric structure. (**C**) The 2D average image of the eNP/octamer 1/0.5 complex (average of 493 particles). (**D**) The 2D average image of the eNP/H3-H4 1/2 complex (average of 412 particles). The 2D average images of eNP (**E**) and of the eNP/H2A-H2B complex (**F**) (21). Bar represents 200 Å in A and 100 Å in (C–F).

To explore how the chaperone and the histone octamers or the H3-H4 tetramers interact to form the high molecular weight complexes described earlier in the text, we resorted to EM. The complexes were formed, negatively stained as described in the ‘Materials and Methods’ section and observed under the electron microscope (Figure 7A). Both the eNP/octamer (1/0.5) and eNP/H3-H4 (1/2) complexes appear as similar football-like particles (Figure 7A and B). After image classification and averaging, the average images of the two complexes confirm the presence of an apparent 2-fold symmetry (Figure 7C and D), their general dimensions being ∼165 Å long and ∼95 Å wide. These structures are much longer than those of eNP (75 Å; Figure 7E) and the eNP/H2A-H2B complex (115 Å; Figure 7F) previously described (21). The latter two structures have a cup shape, and their 3D reconstruction together with the docking of the atomic structures of NP and H2A-H2B point to pentameric NP interacting through its distal face with five H2A-H2B histones (21). As compared with the atomic structure of the NP core (60 Å wide) (11), the wider oligomeric assemblies observed by EM might be due to the presence of the flexible tails and/or the interaction with histones, although negative staining could also contribute to flattening the disordered NP regions. The size and the general shape of the eNP/octamer and eNP/H3-H4 complexes (Figure 7C and D) strongly suggest the presence in these particles of two NP pentamers facing each other and trapping the basic ligands in the central part of the particle through their flexible C-terminal domains that contribute to build the distal protein pole (Figure 7F) (21). The sedimentation velocity analysis have rendered a MW of 307 kDa for the eNP/octamer complex, and this value fits well with a 2/1 eNP/octamer stoichiometry (315 kDa) suggested by the EM analysis. Regarding the composition of the eNP/H3-H4 complex, the stoichiometry estimated from 2D electrophoresis suggests that it contains 2 eNP pentamers and 4 H3-H4 dimers, which at least must form one tetramer, in agreement with the similar average images of both types of complexes.

### NP-bound histone octamers adopt a compact conformation

**Figure 8.**
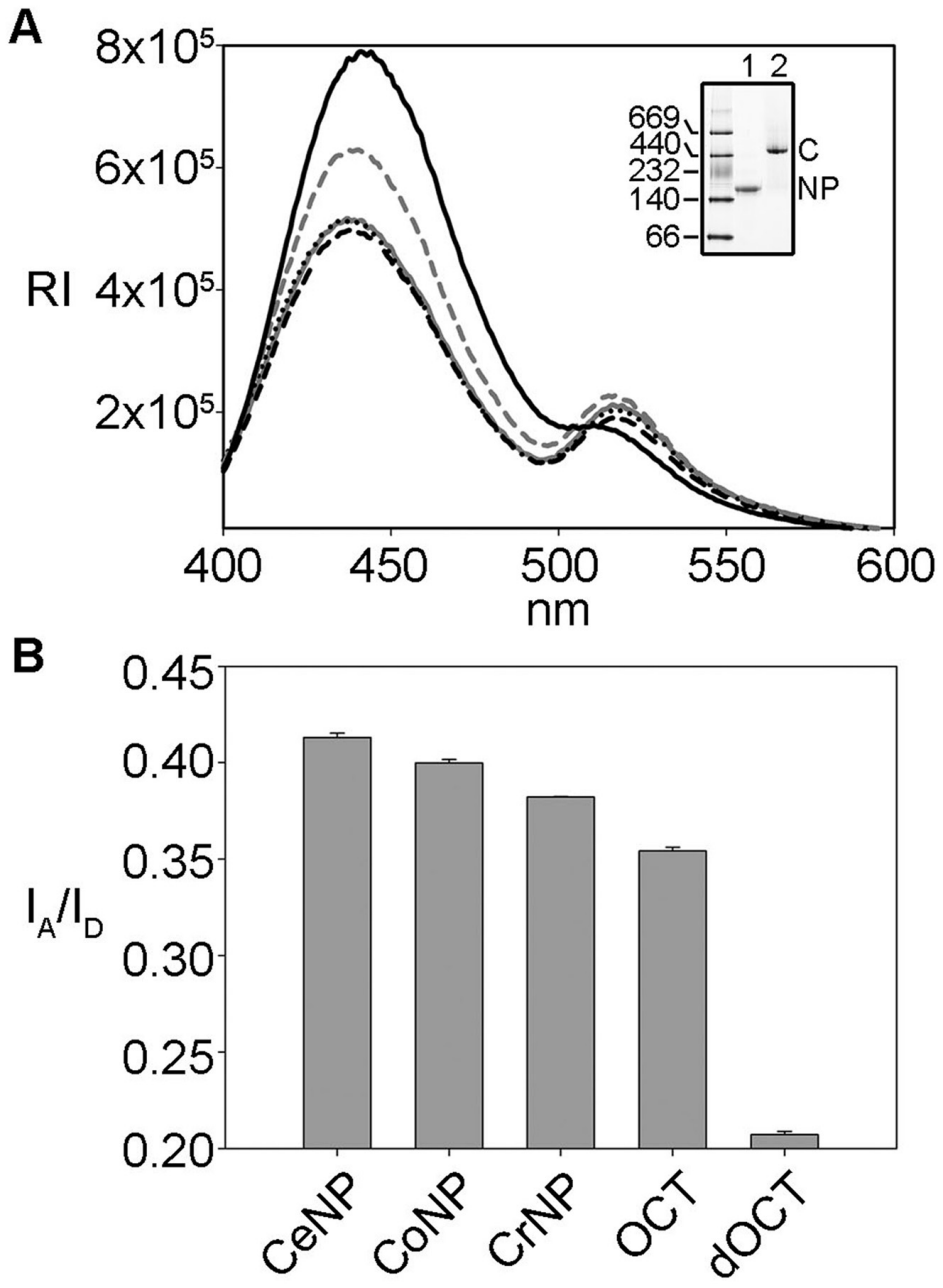
NP-bound octamer adopts a compact conformation. FRET analysis of NP/octamer complexes. (**A**) Emission spectra of eNP/octamer 1/1 (gray solid line), oNP/octamer 1/1 (dotted black line), rNP/octamer 1/0.5 (dashed black line), octamer in 2 M NaCl (gray dashed line) and octamer in 0.24 M NaCl (black solid line). Excitation wavelength was 342 nm. (**B**) Comparison of fluorescence energy transfer of the samples shown in (A), expressed as the ratio of the emission at 519 and 442 nm. Inset: 4–16% Native–PAGE of eNP/Alexa-labeled octamer complex. 1,eNP control; 2, eNP/(H4T71C - H2BT112C) octamer 1/0.5.

To test whether eNP-bound histone octamers adopt a compact oligomeric conformation similar to that found in 2 M NaCl, we performed FRET experiments using fluorescent labeled histones. First, we reconstitute the octamer using H4T71C-Alexa 350 and H2BT112C-Alexa 488 as described (28), and mix it with different native and recombinant NP species. The suitability of FRET to probe the conformation of the histone core octamer is well documented (28,44). Native PAGE demonstrates that histone labeling does not affect NP/octamer complex formation (Figure 8A, inset). We verified that true FRET was observed by measuring the spectra in the absence of acceptor with donor only and vice versa (data not shown). Due to the two-fold symmetry of the histone octamer particle, energy transfer can occur between two donor-acceptor pairs (H4T71C - H2BT112C) if they are separated by a distance close to or shorter than R_o_, in our case 50 Å (44). As expected from a FRET process, in the presence of all NP variants or 2 M NaCl the fluorescence intensity of the donor decreases and concomitantly that of the acceptor increases on exciting the donor (Figure 8A). For a better comparison of the different samples, the fluorescence intensity ratio at 519 and 442 nm was used to follow FRET under different experimental conditions (Figure 8B). The values corresponding to the NP/octamer complexes and to the octamer in 2 M NaCl are similar and two times higher than that of the dissociated octamer in 0.24 M NaCl. Therefore, FRET experiments suggest that NP-bound histones adopt a conformation similar to the octamer stabilized in high salt concentration, even under conditions that favor its dissociation. Moreover, this conformation is also similar regardless of the NP variant (natural or recombinant) used.

## DISCUSSION

NP has been previously characterized as a H2A-H2B histone chaperone with an essential role in chromatin decondensation on oocyte fertilization (3,7,54). Among histone chaperones, some preferentially bind either histones H3-H4 or H2A-H2B while others can interact with more than one histone ligand. In this context, it has been demonstrated that NP interacts with linker histones H5 and H1 (22), sperm basic proteins (SSBP) (22) and H2A-H2B dimers (21,49,55). Our present data show that NP can also bind H3-H4, as described for Nap1, which binds both H2A-H2B and H3-H4 *in vitro* whereas *in vivo* seems to interact preferentially with H2A-H2B (56), and FACT, which interacts with H3-H4 and H2A-H2B during the transcription process (57). The specificity of histone chaperones may be regulated through direct competition between their basic partners, or by temporal and spatial modifications of their chaperone activity *in vivo* (58). The ability of histone chaperones to interact with different histone partners could also explain their essential role in the protein network required to remodel chromatin (6,59). Despite their importance in chromatin dynamics, a deeper understanding of the specificity and mechanism of histone chaperones requires the analysis of complexes between the chaperone and different types of histones, the aim of this study using NP.

### NP binds differently distinct histone partners

NP interacts *in vitro* with H3-H4, regardless of the dimeric or tetrameric state of these hisones, as it has been recently shown for CAF-1 that binds both H3-H4 dimers and tetramers (60,61). In contrast, other histone chaperones preferentially bind H3-H4 dimers (Asf-1 and DAXX) (62–64) or tetramers (Vps75, Nap1 and Rtt106) (65,66). In this context, an interesting finding of this study is that the resulting NP complexes with H3-H4 dimers and tetramers are different. Although the molecular mass of the complexes between NP and H2A-H2B or H3-H4 dimers is similar, the interaction with H3-H4 tetramers gives rise to a significantly larger assembly. Thus, one remarkable difference between the complexes that NP forms with histone dimers (H2A-H2B or H3-H4) or tetramers is the number of molecules that build the assembly and their spatial distribution. The average image obtained for the eNP/H3-H4 complex suggests the presence of two chaperone pentamers that face each other and trap the histone ligand at the center of the ellipsoidal structure (Figure 7D), in stark contrast to the eNP/H2A-H2B assembly that contains five histone dimers interacting with the distal face of one chaperone pentamer (Figure 7F) (21). Binding specificity is a signature characteristic of histone chaperones (6) that varies among the different histone chaperone families (67–69). They can be grouped according to their ability to interact with (i) a wide variety of core and linker histones (NASP and NP); (ii) both types of core histone complexes H2A-H2B and H3-H4 (Nap1 and FACT); (iii) only H3-H4 (Asf-1) or H2A-H2B (Nucleolin); and (iv) specific H3 (CAF-1, DAXX, DEK, HIRA, Rtt106, HJURP) or H2A (CHZ, APLF) variants. Our data suggest that NP displays the promiscuity shown by other histone chaperones (70), as it binds more than one histone type. It should also be kept in mind that the affinity of a chaperone for different histones can vary several orders of magnitude (28,54,60,71,72) and might be modulated by post-translational modifications of both components, as previously reported for NP (54), and/or interactions with different cofactors or others histone chaperones (58,59,73–75).

A second finding of this study is that the structure of the assembly formed by NP and H3-H4 tetramers or histone octamers is similar. Regarding the eNP/H3-H4 complex, a simple hypothesis that might account for its formation is that the interaction between NP-bound H3-H4 dimers could stabilize the complex. However, the stoichiometry of the high molecular weight eNP/H3-H4 complex has been estimated to be ∼1 eNP pentamer/2 H3-H4 dimers, and not 1 eNP/1 H3-H4 dimer. The previously mentioned hypothesis could still be compatible with the experimental stoichiometry if the complex formed by two NP pentamers and a H3-H4 tetramer could additionally bind two histone dimers or one tetramer. This ability would depend on the availability of binding sites in the chaperone, which as judged by the overall similarity of the EM images of the eNP/H3-H4 and eNP/histone octamer complexes would suggest that this possibility might happen. Deciphering which of these alternatives takes place will require a careful analysis of the 3D reconstruction of these complexes.

The aforementioned similarity also suggests that binding of histone octamers could be, at least initially, driven by the recognition of the H3-H4 component, which would trigger the subsequent interaction of the negatively charged and flexile distal face of the chaperone with the other core histones. This is further supported by the similar size and apparent 2-fold symmetry of the two complexes, in which the H3-H4 tetramer(s) and histone octamer are bound in a similar fashion between the two opposing NP pentamers, with their pseudo 2-fold symmetry axis roughly perpendicular to the long axis of the particles. Formation of recombinant NP/octamer complexes has been reported *in vitro* (11,29,45), albeit with significantly different stoichiometries, suggesting that the ability to bind histone octamers could be a characteristic of proteins of the NP/nucleophosmin family (14). Our data also demonstrate that the important features that allow NP to bind differently the various histone oligomerization states do not rely on NP hyperphosphorylation (17,18), a post-translational modification that significantly enhances its chromatin decondensation activity (3,16,22). Phosphorylation most likely modulates the affinity of the chaperone for its basic partners, as found for the interaction with H2A-H2B dimers (54).

### Biological relevance

The interaction of NP with both types of nucleosomal histones *in vivo* has been a matter of controversy. It was first suggested that NP could interact with H2A-H2B and with variants of H3 (55). The H3 ligand was identified as a H2A variant in a later study, and therefore it was proposed that H3-H4 were associated with NASP (N1/N2) and H2A-H2B with NP (49). In a subsequent work, a very minor histone component, identified as H4, was found bound to NP in *X. laevis* oocytes (50). One of the reasons that could, at least partially, explain these contradictory data is that NP-core histone complexes could be weak or transiently assembled, and thus their final composition would depend on the experimental conditions used to isolate them. Despite this confusing background, it seems clear that NP preferentially binds H2A-H2B, although it could, as we show herein, also interact with H3-H4. A role of NP in transferring H3-H4 to DNA cannot be discarded and would not be surprising, as it occurs with other histone chaperones that display similar overlapping functions (76,77). Moreover, the ability of NP to recognize chromatin H3-H4 tetrasomes may provide the critical orientation for the further assembly of its bound H2A-H2B dimers into proper nucleosome complexes, similarly to what it has been proposed for FACT (57).

It is difficult to extrapolate the role that the NP-octamer interaction observed *in vitro* might have *in vivo.* NPM1, a member of the NP/nucleophosmin family present in oocytes, embryonary and adult cells that has been involved in ribosome biogenesis and association with pre-ribosomal RNP particles DNA (23,78), also forms complexes with H3-H4 and histone octamers *in vitro* (13,45,78). Although this shared ability could suggest that these assemblies might display a specific biological function in the nucleolus of normal cells, their existence *in vivo* remains to be proved. Binding and stabilization of H3-H4 tetramers and histone octamers by NP preserves the natural interactions of histones within the tetrasome and nucleosome, respectively, which it could be used to increase the efficiency of histone transfer onto DNA (28,67,79). A question that remains as yet unanswered is how the NP-histone complexes dissociate during histone deposition onto DNA or transfer to another histone chaperone. Complex dissociation is a prerequisite for histone exchange and most likely requires a competition between DNA and histone chaperones or between different histone chaperones for the basic ligands. This competition would destabilize the complex generated by wrapping the basic ligands with the acidic tails of the chaperone, thus facilitating histone transfer. This would mean that histone exchange could be guided by a change in the relative affinity of histones for histone chaperones and DNA, which might be controlled by post-translational modifications (58).

### Model for the interaction of NP with histones

Two different models have been proposed for the interaction of NP with histones. The first one involves the lateral face of the protein in histone binding (45). Through this protein region, a NP pentamer would first interact with H2A-H2B dimers, and the subsequent binding of H3-H4 tetramers would mediate association of two pentamer-histone dimer complexes. The NP decamers would therefore create on their lateral face a binding region for H2A-H2B dimers and H3-H4 tetramers that could assemble into histone octamers on the chaperone (45). An alternative model has been suggested for the interaction of NP with H2A-H2B dimers, in which histones bind to the distal face of the chaperone, a flexible and negatively charged region able to accommodate five histone dimers per NP pentamer (21). This model has been proposed based on the reconstruction by EM of the 3D structure of the NP/H2A-H2B complex, therefore being the unique direct experimental evidence revealing the mode of interaction of NP and H2A-H2B histones (21). Other experimental techniques, such as small-angle X-ray scattering (SAXS), have not unambiguously revealed whether the binding region was formed by the distal or lateral faces of the chaperone (54). Data presented herein indicate that the distal face of NP forms the interacting surface with both H3-H4 tetramers and histone octamers and demonstrate that they form large complexes in which two NP pentamers face each other through their intrinsically disordered, distal face and trap in the center of the structure the basic ligands. Interestingly, our model is consistent with recently reported FRET experiments (45), as the distance between the acceptor-donor pair, located at Cys42 within the A1 loop, estimated with our EM average image and with the model proposed by Platonova (45) is similar, i.e. ∼80 Å.

Another important difference between these models is the stoichiometry of the resulting complexes: 5 octamers per NP decamer (13) or 1 octamer per 2 NP pentamers. Therefore, in the previous model (13), as we have recently found for the interaction of NP with H2A/H2B dimers, complex formation follows the symmetry of the chaperone, whereas in the model presented here, it uses the symmetry of the H3-H4 tetramer either free in solution or forming part of the histone octamer. In both models, electrostatic interactions would favor association of all core histones to NP, resulting in effective charge neutralization, whereas steric constraints imposed by chaperone and histone architecture could confer additional specificity to the histone binding mode. In particular, the difference in surface-exposed residues and oligomerization state between H2A-H2B and H3-H4 might be behind the different stoichiometry of the complexes. Dimeric Nap1, Vps75 and Rtt106 bind H3-H4 tetramers (65,66), whereas CAF-1 does it as a trimer (60,73) in solution. These complexes are stabilized by interactions between the histone tetramer and two identical (65,66) or different (60) chaperone subunits. The pentameric structure of NP might allow binding of histone dimers, tetramers and octamers, due to the large negatively charged and adjustable distal face of the protein, which unlike other histone chaperones can hold ligands of different size. If we consider that the size, amount of positively charged residues and/or exposed hydrophobic patches of a histone dimer can be ‘neutralized’ by a chaperone subunit, then a stoichiometry of 1 NP subunit/1 histone dimer or a particle with a 5-fold symmety would be obtained, as experimentally observed for H2A-H2B (21). However, when the histone ligand is a tretramer or octamer, several chaperone subunits must collaborate to stably bind the ligand, generating a complex with an apparent 2-fold symmetry. Therefore, this change in symmetry is driven by differences in the histone ligand, which regardless of its identity interacts with the same region of the chaperone, the distal face. This interpretation is supported by the fact that deletion of the C-terminal, intrinsically disordered domain of NP weakens the interaction with histones (Supplementary Figure S3) (21) and its chromatin-decondensation activity (80). This domain has previously been proposed to exert a stabilizing role in NP-histone complex formation (29) and to increase 10 and 10 000 times the affinity of recombinant NP for H2A-H2B and H1, respectively (54). The A1 tract, a short loop with six acidic residues, acts synergistically with the C-terminal domain to bind core histones forming large complexes, similar to those seen in this study (45), and is involved in the decondensation activity of NP (81).

In summary, the study presented herein demonstrates the diverse ways used by a histone chaperone to bind its basic partners. This experimental observation strongly suggests that NP can form stereospecific complexes with histones, beyond the electrostatic component of the interaction, as previously postulated (70). That would explain how the chaperone could take part in so many chromatin organization and dynamic related pathways. Different modes of histone recognition, besides pursuing the task of preventing the undesired unspecific aggregation may also provide a mean for efficient deposition of histone components during sequential nucleosome assembly. The use of the same chaperone structural region to accommodate all types of histones could favor histone exchange, an essential process for nucleosome dynamics and histone transfer within the chaperone network. Therefore, this study provides an example of the diverse histone-binding modes of NP that might reflect the variety of mechanisms that histone chaperones use to pursue their functions.

## SUPPLEMENTARY DATA

Supplementary Data are available at NAR Online.

## FUNDING

Ministerio de Ciencia e Innovación [Grants BFU2010-15443 to A.M. and BFU2010-15703 to J.M.V.]; the Universidad del País Vasco and Gobierno Vasco [Grant IT709-13]; and Natural Sciences and Engineering Research Council of Canada (NSERC) [Grant 46399-2012 to J.A.]. Funding for open access charge: Ministerio de Ciencia e Innovación [Grant BFU2010-15443].

*Conflict of interest statement.* None declared.

## Supplementary Material

Supplementary Data
