# Supplementary material for: The intrinsically disordered distal face of nucleoplasmin recognizes distinct oligomerization states of histones
Source: Nucleic Acids Res. 2013 Oct 8;42(2):1311–25. doi: 10.1093/nar/gkt899 (PMC3902905; doi:10.1093/nar/gkt899)
Supplement: Supplementary Data [file supp_42_2_1311__index.html]

The intrinsically disordered distal face of nucleoplasmin recognizes distinct oligomerization states of histones — The intrinsically disordered distal face of nucleoplasmin recognizes distinct oligomerization states of histones — Supplementary Data 

# The intrinsically disordered distal face of nucleoplasmin recognizes distinct oligomerization states of histones

## Supplementary Data

files

**Files in this Data Supplement:**

- Supplementary Data - pdf file
