# Supplementary material for: The intrinsically disordered distal face of nucleoplasmin recognizes distinct oligomerization states of histones
Source: Nucleic Acids Res. 2013 Oct 8;42(2):1311–25. doi: 10.1093/nar/gkt899 (PMC3902905; doi:10.1093/nar/gkt899)
Supplement: Supplementary Data [file supp_gkt899_nar-02130-x-2013-File010.pdf]

SUPPLEMENTARY DATA

The intrinsically disordered distal face of nucleoplasmin recognizes distinct oligomerization states of histones

Isbaal Ramos, Noelia Fernández- Rivero, Rocío Arranz, Kerman Aloria, Ron Finn, Jesús M. Arizmendi, Juan Ausió, José María Valpuesta, Arturo Muga, and Adelina Prado

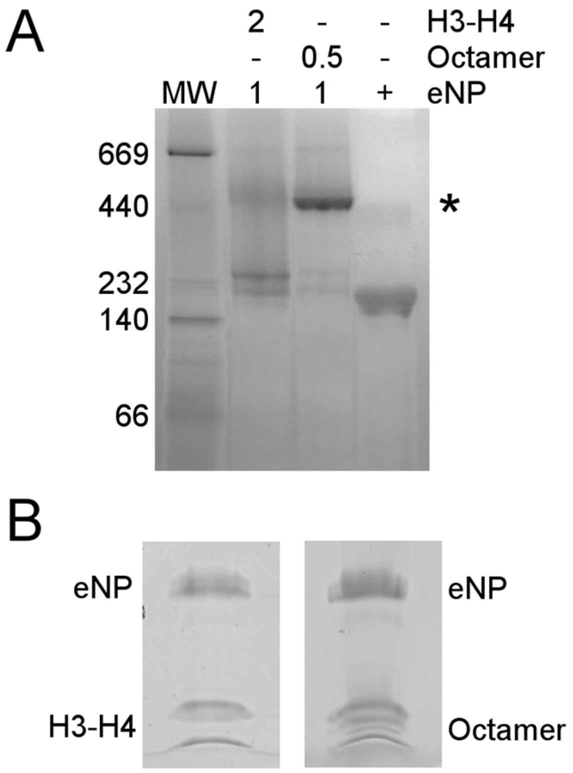

Figure S1

Figure S1. Two-dimensional electrophoresis of the eNP/H3-H4 (1/2) and eNP/histone octamer (1/0.5) complexes. A) First dimension: Native 4.16% PAGE of eNP/H3-H4 (1/2) and eNP/histone octamer (1/0.5) complexes formed in 150 mM NaCl, 25 mM Tris-HCl, 2mM $MgCl_2$ (pH 7.5). Proteins were negatively stained with Zn-Imidazole. The high molecular bands (indicated by an asterik) were excised, boiled in SDS sample buffer for 10 min and analyzed by B) 12.5% SDS-PAGE (second dimension). Proteins were stained with Coomasie Brilliant Blue. eNP, H3-H4 and histone octamer bands are indicated. The eNP/histone molar ratios of these complexes estimated by densitometry were 1/2.1 and 1/0.54 for the eNP/H3-H4 and eNP/octamer complexes, respectively.

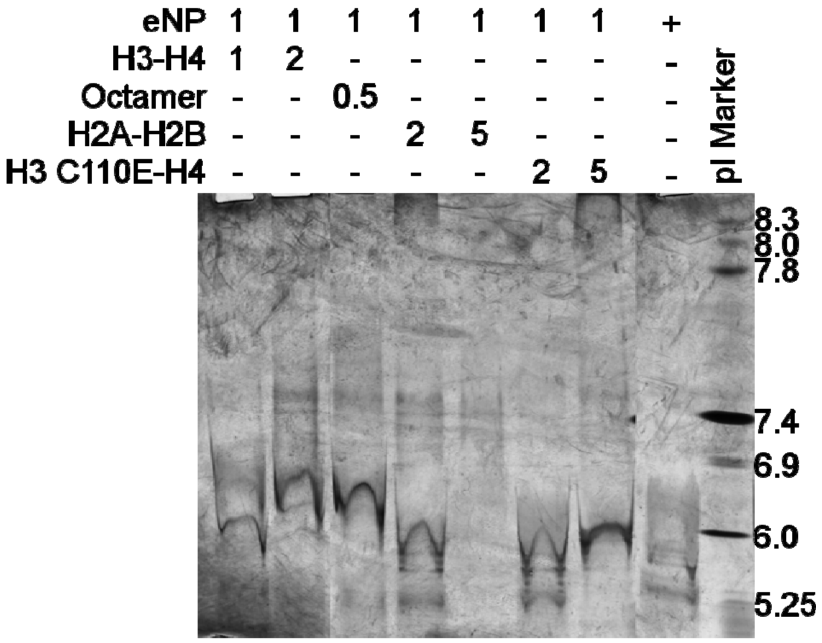

Figure S2

Figure S2. Isoelectric Focusing of different eNP/Histone complexes. Samples containing the indicated eNP/histone molar ratios were incubated 1 h at room temperature in 150 mM NaCl, 25 mM Tris-HCl (pH 7.5), 2 mM $MgCl_2$. IEF-5% Native-PAGE (Invitrogen) of eNP (4 μM) and eNP/histone complexes was run at 4ºC for 2.5 h. After 30 min at room temperature in 12% TCA, the gel was stained with Coomassie Brilliant Blue.

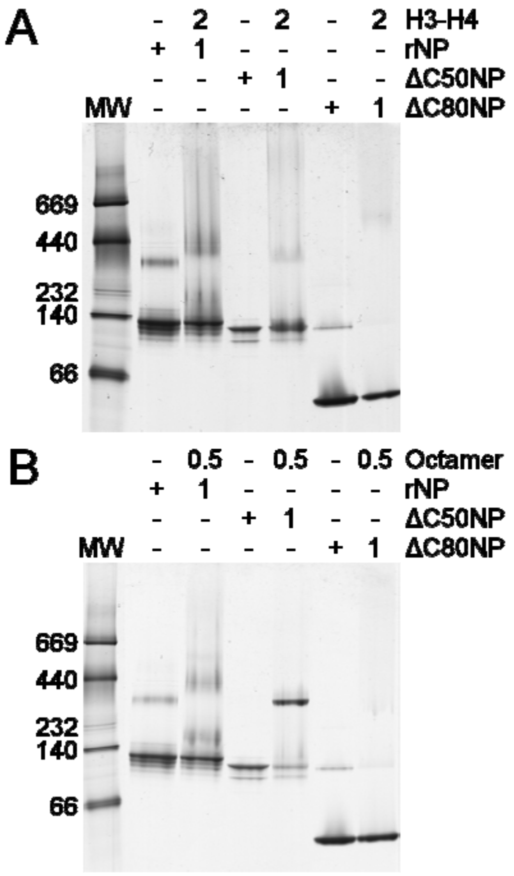

Figure S3

Figure S3. Interaction of different variants of recombinant NP with H3-H4 and histone octamers at 240 mM NaCl. Mixtures of rNP, ΔC50NP, or ΔC80NP (3 μM) and histones were incubated and analyzed by Native PAGE as described in the Methods section. A) Recombinant nucleoplasmin/H3-H4 complexes. The recombinant variants of NP were mixed with histones at the indicated molar ratios, and analyzed by Native PAGE. As a control, the same NPs were run in the absence of basic ligands. B) Recombinant nucleoplasmin/octamer complexes. The same NP variants were mixed with histone octamers at the indicated molar ratios, and analyzed by Native PAGE. The appearance of a high molecular weigh band in the samples corresponding to rNP and ΔC80NP in the absence of histones, suggests that a small proportion of these recombinant variants could associate to form decamers under our experimental conditions. Supporting this interpretation is the fact that this behavior is observed neither for ΔC50NP, most likely due to electrostatic repulsions between the exposed polyGlu tracts, nor for hyperphosphorylated eNP. Upon complex formation with either H3-H4 or histone octamers these bands disappear, indicating that mainly NP pentamers are involved in histone binding.

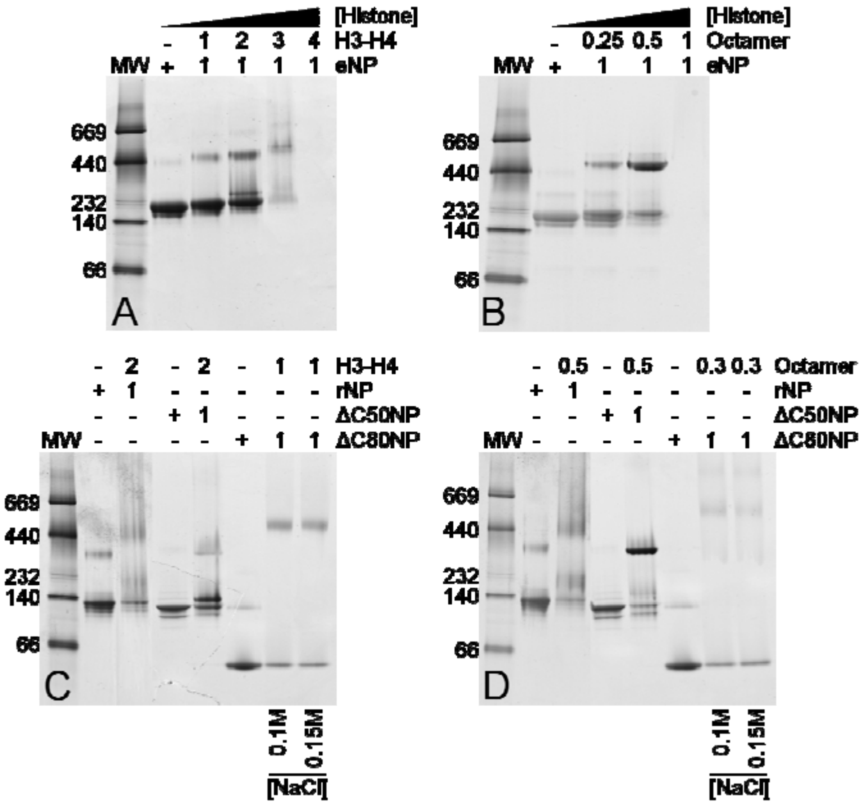

Figure S4

Figure S4. Effect of the ionic strenght on the interaction of eNP and different variants of recombinant NP with H3-H4 and histone octamers. Mixtures of rNP, ΔC50NP, or ΔC80NP (3μM) and histones were incubated in buffer containing 150 mM or, when indicated, 100 mM NaCl (panels C and D), and analyzed by Native PAGE. Titration of eNP with H3-H4 (A) or histone octamers (B). The triangle shows the increase in histone concentration. Interaction of the different recombinant NP variants with H3-H4 (C) or histone octamers (D). The eNP/histone molar ratios (eNP pentamer/histone dimer or octamer) are indicated above each gel. The high MW bands in the ΔC80/H3-H4 and ΔC80/octamer samples at 100 and 150 mM NaCl in panels C and D, indicate that this truncated NP variant can interact with histones under these experimental conditions. However, these complexes might be different from those observed with rNP and ΔC50NP, since they do not shown the expected decrease in molecular mass if we assume that they are formed by two NP pentamers and two H3-H4 tetramers or one octamer. This difference in molecular mass, associated with the deletion, is clearly observed for the complexes of rNP and ΔC50NP with either H3-H4 or histone octamers.

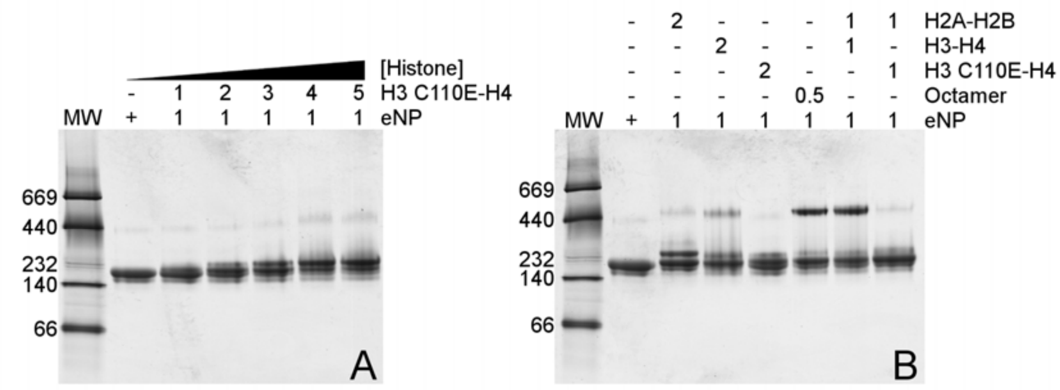

Figure S5

Figure S5. Titration of eNP with different histones at 150 mM NaCl. Native PAGE of A) eNP/ H3C110E-H4 complexes. The triangle shows the increase in histone concentration. B) Comparison of different eNP/histone complexes. The two samples at the right of the gel were prepared by diluting while mixing both types of nucleosomal histones from stock solutions in 2 M NaCl, in the presence of NP. The molar ratio of the different mixtures is indicated above each gel.
